# Blockade of Cochlear NMDA Receptors Prevents Long-Term Tinnitus during a Brief Consolidation Window after Acoustic Trauma

**DOI:** 10.1155/2007/80904

**Published:** 2008-02-06

**Authors:** Matthieu J. Guitton, Yadin Dudai

**Affiliations:** ^1^Department of Neurobiology, The Weizmann Institute of Science, Rehovot 76100, Israel; ^2^Molecular Endocrinology and Oncology Research Center, Laval University Medical Center (CHUL), Quebec City, QC, Canada G1V 4G2; ^3^Faculty of Pharmacy, Laval University, Quebec City, QC, Canada G1K 7P4

## Abstract

Tinnitus, the perception of sound in the absence of external acoustic stimulation, is a common and devastating pathology. It is often a consequence of acoustic trauma or drug toxicity. The neuronal mechanisms of tinnitus are neither yet fully understood nor are effective treatments available. Using a novel behavioral paradigm for measuring tinnitus in the rat based on tone-guided navigation, we show here that the development of long-term noise-induced tinnitus, the most prevalent and clinically important form of human tinnitus, can be abated by local administration of the NMDA antagonist “ifenprodil” into the cochlea in the first 4 days following the noise insult but not afterwards. This suggests that long-term tinnitus undergoes a consolidation-like process, resembling the ontogeny of items in long-term memory. Furthermore, this finding paves the way to potential therapeutic strategies for the prevention of chronic tinnitus once the noise insult had taken place.

## 1. INTRODUCTION

Tinnitus, the perception of sound in the absence of external acoustic stimulation, is a widespread pathology and affects around 10% of the adult population [1–4]. It is commonly the result of overexposure to noise or overconsumption of drugs such as salicylates. During the past decades, clinical studies, however, consistently reported noise overexposure as the main cause of tinnitus in human [1, 2, 5, 6]. Despite the strong alteration of the quality of life of patients suffering from tinnitus and its impact on public health systems, and despite increasing knowledge on the molecular mechanisms involved [7–10], no efficient cure is currently available [4, 6, 9].

Given the high prevalence and the suffering involved, the neurobiology of tinnitus is of great importance. It bears theoretical interest as well. What is it that alters a perceptual system to experience a phantom percept? Is this a form of runaway plasticity, and can it be blocked? Are its mechanisms shared with those that subserve memory? Evidence has been accumulated to implicate neuroplasticity in tinnitus, including a role for cochlear N-methyl-D-aspartate (NMDA) receptor [7, 11, 12].

Most studies use salicylate-induced tinnitus as a model. This form accounts, however, for only a minor fraction of human tinnitus; acoustic trauma is much more prevalent [2, 5]. Further, whereas salicylate-induced tinnitus is reversible, noise-induced tinnitus is frequently chronic. The mechanisms of noise-induced tinnitus are hardly understood. A particularly important question: once the noise insult took place, is there still an opportunity to abate tinnitus?

To achieve progress towards the aforementioned goals, an animal model is needed. The first behavioral model in the rat was introduced by Jastreboff et al. [13]. They used noise-controlled conditioned suppression of drinking, and showed that rats treated with salicylate are less likely to stop drinking when the noise is turned off. This has been taken to indicate that the salicylate-treated rats still hear the sound in its absence. This imaginative protocol, however, requires extensive training, utilizes footshock that may introduce confounding factors, and involves intense water deprivation. We have set out to develop a new behavioral test for tinnitus in the rat, based on navigation to a tone in a water T-maze (WTM). Based on the behavior of salicylate-treated rats, we define criteria for identification of tinnitus in the WTM, and use them to identify noise-induced tinnitus. Here, we report that the induction of both salicylate-induced and noise-induced tinnitus can be blocked by the local cochlear application of ifenprodil, an antagonist of the 2B subunit of the NMDA receptor (NR2B), a molecule which is implicated in long-term potentiation and behavioral plasticity in the mammalian brain [14–18]. We further report that this NR2B-dependent process undergoes consolidation, during which the development of long-term tinnitus can be prevented by an NR2B blocker. Our data hence demonstrate a consolidation window in trauma-induced plasticity in a peripheral sensory system, and point to a potential method to abate the outcome of the sensory trauma.

## 2. MATERIALS AND METHODS

### 2.1. Animals

Rats (Wistar males, ~60-day old, 250–380 g, total *n* = 154) were caged individually at 22 ± 2°C in a 12-hour light/dark cycle. Water and food were available ad libitum. All experiments were approved by the Weizmann Institute of Science Animal Care and Use Committee. The repartition of the animals in the different experimental groups is detailed in Table 1.

### 2.2. Behavioral paradigm

**Figure 1:**
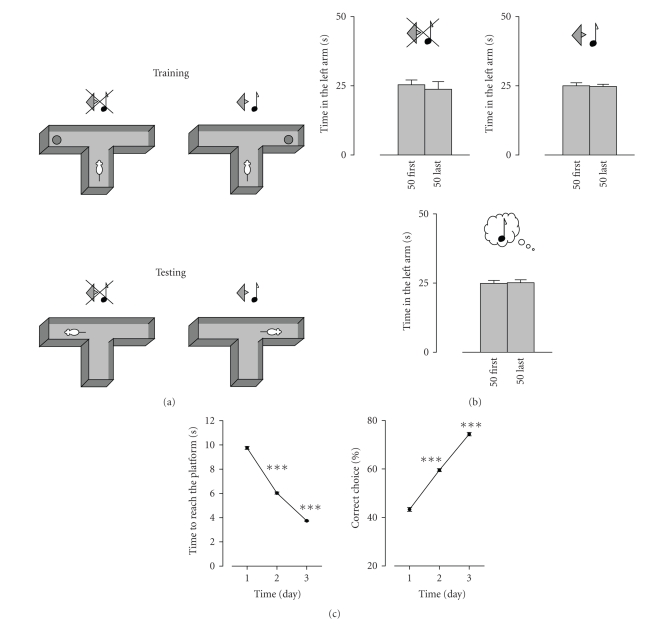
*The behavioral paradigm*. (a) *Schematic description of the task*. Rats were conditioned to associate the location of a submerged platform in a water T-maze (WTM) with the absence or presence of a tone. When the tone was present, the platform was in one arm (tone arm), whereas when the tone was absent, the platform was in the opposite arm (no-tone arm). The sidearm-platform-tone permutations were counterbalanced between subjects within each treatment group to control for potential side preference. In the test, conducted 2 weeks after the end of training, the rat was placed in the maze for 100 seconds in the absence of the platform. The arm entered first by the rat and the time spent in each arm in the first and in the last 50 seconds of the test were recorded. (b) *Lack of arm preference in the WTM test in the absence of conditioning.* Animals were tested in the absence of the tone (upper panel, *n* = 8), in the presence of a 10 khz tone (middle panel, *n* = 8), or in the absence of the tone after salicylate treatment at a dose established to induce tinnitus (300 mg/kg/day for 4 days, tested 2 hours after the last administration, lower panel, *n* = 8). (c) *Learning curves of the acquisition of the tone-platform association*. Acquisition of the tone-platform association, expressed in time to reach the platform and in percent time spent in the correct arm, each averaged per training day (*n* = 124, ***P < .001 compared to the previous training day).

We used a place-tone conditioning paradigm, in which the rat is conditioned in a water T-maze (WTM, see Figure 1) to associate a tone with the presence of a submerged escape platform in one of the arms (tone arm), and the absence of that tone with the presence of the platform in the opposite arm (no-tone arm). The arm-platform-tone permutations were counterbalanced between subjects in the experimental design to eliminate potential side preference. Rats with tinnitus were expected to always behave as though the tone was present, even in its absence. All arms of the WTM were made of black Plexiglass. The starting arm was 25-cm long and the two identical lateral arms 40 cm long each. All arms were 15-cm wide and 60-cm high, filled with water to a level of 24 cm. Water temperature was 21 ± 1°C. A sliding door was installed before the maze decision point. A submerged platform (12-cm diameter, 23 cm height) was placed in one of the arms. The tones used were 10 kHz or 6 kHz (as indicated under results) continuous pure tones, 45 dB SPL, delivered from above by a speaker (High Performance 3” Tweeter, Best-Star). In the conditioning phase, rats received 1 session/day for a total of 3 days. Each session consisted of 12 trials (3 *no-tone* + 3 *tone* alternating twice in that order). The platform was positioned at the end of one arm during the tone presentation and at the end of the opposite arm during the no-tone period. The rat was placed in the starting arm for 5 seconds before the opening of the door. The door was closed after the entrance of the rat to the lateral arm. When applicable, sound onset coincided with the placing of the rat in the starting arm and continued to overlap the first 5 seconds after the rat reached the platform. When the rat located the platform, it was allowed to stay on it for 30 seconds before being placed back in the starting arm (beginning of the next trial) or in the home cage (after the last trial).

The following two parameters were used to quantify conditioning in the course of *training*: * time to reach the platform* (averaged over the 12 trials of each session), and *correct decision* (percentage of correct responses over 12 decisions made in a session). The test protocol consisted of a single trial performed in the absence of the platform. Except when otherwise specified, rats were tested in the absence of the tone. When rats were tested with the tone, the tone remains on for entire duration of the test trial. The rats were placed in the starting arm 5 seconds before the opening of the door. The duration of the trial was 100 seconds from the entrance of the rat into the lateral arm. After this, the rat was placed back in its home cage. To quantify memory in the *test* session, we recorded the *time spent by a rat in each arm.* This is presented in Section 3 as time spent in the indicated arm in the first 50 seconds and in the last 50 seconds, respectively. This breakdown into early and late test period was done to allow detection of possible alteration in behavior during the test itself. We also recorded the first arm-selection made by each individual rat. This was done to supplement at the group level the observations made of the behavior of the individual rats. These data are presented only when the size of the resulting subgroups was large enough to allow statistical analysis.

### 2.3. Noise overexposure

This was performed in an acoustic chamber (Controlled Acoustic Environments, Industrial Acoustics Company Inc., New York, NY, USA). Rats were exposed to a 6 kHz, 130 dB SPL pure-tone for 15 minutes. Optimal tone detection in the rat, as assessed by electrophysiological recordings of auditory thresholds, is 10 kHz [7]. In previous morphological and electrophysiological studies, maximum damage was observed in the tonotopic cochlear area coding for 10 kHz following an acoustic overexposure of a pure-tone of 6 kHz [19]. Sound insult at 6 kHz was therefore selected to maximize the occurrence of tinnitus of a frequency of 10 kHz. The tone was delivered after an analog amplification (MA 430 Power Amplifier, Inkel, Seoul, South Korea) via high-power speakers (XD 120, Eighteen Sound, Reggio Emilia, Italy). Rats were anesthetized before the noise overexposure by IP injection of 0.3 mL/kg sodium pentobarbital at 6% (CTS Chemical Industries Ltd., Tel-Aviv, Israel). All sound levels were measured and calibrated using a Brüel and Kjaer microphone (BK Precision 732, Brüel and Kjaer, Norcross, Ga, USA).

### 2.4. Surgical procedures

Rats were anesthetized with IP injection of 0.3 mL/kg sodium pentobarbital at 6% and operated under aseptic conditions. The surgical protocol to place Gelfoam (Gelita tampon; B. Braun Medical, Melsungen, Germany) on the round window of both cochleae was as described previously [7]. After exposition of the cochlea via aposterior approach of the right bulla, Gelfoam, soaked with 2.5 *μ*L of artificial perilymph containing the appropriate drug, was applied to the round window of the cochlea. The bulla was closed with dental cement, and the surgical wound was sutured. The same procedure was replicated in the other ear.

### 2.5. Drugs

Salicylate, ifenprodil, and metachlorophenylpiperazine (mCPP) were purchased from Sigma (Sigma-Aldrich, St. Louis, Miss, USA), 6,7-dinitroquinoxaline-2,3-dione (DNQX) was from Tocris (Tocris Bioscience, Avonmouth, UK). Salicylate was dissolved in saline and kept in the dark. The N-methyl-D-aspartate (NMDA) receptor antagonist ifenprodil, specific to NR2B-subunit, was used at a concentration of 10 *μ*M. The *α*-amino-3-hydroxy-5-methyl-4-isoxazole propionic acid (AMPA) receptor antagonist DNQX was used at a concentration of 50 *μ*M. The serotonergic agent mCPP was used at a concentration of 50 *μ*M. Ifenprodil, DNQX, and mCPP were all dissolved in artificial perilymph (AP, in mM: 140 NaCl, 4 KCl, 2 CaCl_2_, 2 MgCl_2_, 10 HEPES, 10 glucose, pH 7.4, and osmolarity, 300 ± 10 mOsm/kg H_2_O). Drug solutions were prepared freshly before each experiment. Neither nystagmus nor apparent dizziness was observed after local application of any of the pharmacological agents at the concentrations we used onto the round window, suggesting a lack of effect on the vestibular function.

### 2.6. Statistical analysis

Behavioral data were analyzed by one-way ANOVA followed by post hoc comparisons (Tukey's test). Data are presented as mean ± SEM. In experiments which involved time, comparisons were made using a two-way ANOVA (group × time, with repeated measures on the last factor) to test group effect, time effect, and group × time interactions, followed by post hoc comparisons (Tukey's test). Proportions of rats experiencing tinnitus in various treatment groups were compared by chi-square test or Mann-Whitney test. In addition, the occurrence of tinnitus over time following pharmacological treatment was assessed using a Wilcoxon test on the time spent in the tone arm.

## 3. RESULTS

### 3.1. The behavioral paradigm and its validation

In the first phase of this study, we developed a novel behavioral paradigm that permits objective determination of tinnitus in the rat, and verified the power of this paradigm to detect tinnitus by the use of salicylate treatment under conditions that are well established to produce tinnitus. We used a place-tone conditioning paradigm, in which rats learn to associate the presence of a tone with the presence of an escape platform in one of the arms (tone arm) of a water T-maze (WTM), and the absence of that tone with the presence of the escape platform in the opposite arm of the maze (no-tone arm). Akin to the reasoning of previous animal protocols intended to detect tinnitus [7, 13], we reasoned that rats with tinnitus, experiencing phantom tone, would always behave as though the tone is present, even in its absence. The tone employed was 10 kHz. This frequency is optimally perceived by the intact rat [7] and characterizes salicylate-induced tinnitus in the rat [7, 20].

Unconditioned rats displayed no arm preference in the WTM, as judged by the time spent in each arm (see Figure 1(b)). This was regardless of whether they were tested in the presence of the tone, in its absence, or in the absence of the tone after being treated for 4 days with salicylate under conditions known to induce tinnitus [7, 20]. Thus, in the absence of the tone, the unconditioned rats spent 25.4±1.7 seconds and 23.7±2.7 seconds in the left arm during the first and the last 50 seconds of the test, respectively. When tested in presence of the 10-kHz sound, the corresponding values were 25.0±1.1 seconds and 24.7±0.8 seconds. Finally, when tested in the absence of the tone, unconditioned salicylate-treated rats spent 24.9±1.1 seconds and 25.1±1.1 seconds in the left arm during the first and the last 50 seconds of the test, respectively. Further, the first arm choice was made randomly: 4 of the 8 of the unconditioned rats selected the left arm as their first choice.

In contrast, in conditioned rats, a clear preference became evident over training for the tone- /no tone-cued arm associations, as manifested in the time to reach the platform and in choice preference (learning curve, Figure 1(c)). Hence, on Day 1 of training, the mean time to reach the platform was 9.8±0.1 seconds and accuracy was 43.3±0.8%, reaching 3.7±0.05 seconds and 74.4±0.7%, respectively, on Day 3 (see Figure 1(c), *n* = 124).

**Figure 2:**
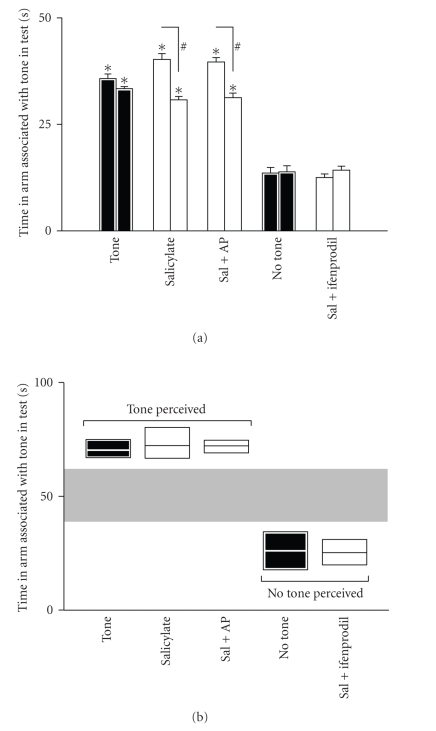
*Induction of tinnitus by salicylate (sal), its blockade by NR2B antagonist (ifneprodil), and establishment of behavioral criteria for tinnitus.* (a)* Time spent in the tone arm during the test*. The time spent by various treatment-groups (identified along the *X*-axis) in the tone arm during the first 50 seconds (left-hand bar in each pair) and the last 50 seconds of the test. Black bars refer to groups of animals which were tested after conditioning without any treatment. The test was performed two weeks after the end of conditioning. Rats tested in the presence of the tone displayed preference for the tone arm, whereas rats tested in the absence of the tone displayed preference for the no-tone arm. Salicylate-treated rats (300 mg/kg/day for 4 consecutive days, the last injection taking place 2 hours before the test) behaved as if they perceive a tone, spending most of their time in the tone arm though the tone was absent. Cochlear application of artificial perilymph (AP) had no effect, but cochlear application of ifenprodil reversed the behavior induced by salicylate (*P < .01 from animals tested in silence, # *P* < .01 from the first 50 seconds window of the same group (*n* = 8 each)). (b) *Definition of the criteria used to designate rats as experiencing tinnitus.* Rats were designated as perceiving a tone in its absence, that is, suffering from tinnitus, if in the WTM test in the absence of the tone they spent in the tone arm not less than 2 SD (*P* > .05) below the mean time spent by untreated conditioned rats, tested in the presence of the tone, in the tone arm. Similarly, treated rats were designated as lacking tinnitus if in the test they spent in the tone arm not more than 2 SD above the mean time spent by untreated conditioned rats, tested in the absence of the tone, in the tone arm (the separation of the two possible behavioral patterns generated according to the aforementioned criteria is emphasized by the grey zone). These criteria were used to designate rats as experiencing tinnitus in the noise-induced tinnitus experiments.

The memory for the association remained robust two weeks after the end of training (see Figure 2). When tested in the absence of the tone two weeks after the end of training, the conditioned rats spent only 13.4±1.2 seconds and 14.1±1.3 seconds during the first and the last 50 seconds of the test, respectively, in the tone arm (see Figure 2(a)). In contrast, conditioned rats tested in presence of the tone displayed significant preference for the tone arm. They spent 35.8±1.1 seconds and 33.4±0.5 seconds in that arm during the first and last 50 seconds of the test, respectively (different from the conditioned rats tested in the absence of the tone in both time windows, *P* < .001, see Figure 2(a)). After salicylate treatment, the rats spent 40.3±1.4 seconds during the first 50 seconds and 30.8±0.8 during the last 50 seconds of the test in that arm (different in both cases from the conditioned animals tested in the absence of the tone, *P* < .001). In addition, despite being in both cases significantly different from the untreated conditioned animals tested in the absence of the tone, the time spent in the tone arm by salicylate-treated conditioned rats was significantly higher during the first 50 seconds of the test (*P* < .01). This might imply that the subjective perception of the cue in salicylate-treated rats is not exactly as in the untreated rats. An alternative hypothesis could be that animals behave differently in the test due to an elevation of their auditory thresholds [7]. In the present protocol, given that animals are tested in the absence of the tone, deaf animals would be expected to have a greater probability to spend time in the no-tone arm. Indeed, the physical absence of the tone would probably combine with the perceptive silence of deafness. This, however, is not the case: following salicylate treatment, animals spent more time in the tone arm. Thus, a contamination of deafness in the interpretation of these data can be ruled out.

Rats treated with salicylate as above, but receiving a local cochlear application of ifenprodil, an NR2B antagonist, performed in the WTM similarly to untreated rats tested in the absence of the tone (Figure 2(a)). They spent only 12.5±0.9 seconds during the first 50 first seconds and 14.3±0.9 seconds during the last 50 seconds of the test in the tone arm (not significantly different from untreated rats). Local application onto the cochleae of the vehicle only had no effect on salicylate-treated rats (Figure 2(a)).

### 3.2. Noise-induced tinnitus

The results obtained above allowed us to establish criteria to identify rats with tinnitus by their behavior in the WTM (see Figure 2(b)). In the aforementioned experiments, two distinct behavioral patterns became apparent: one, “tone”, corresponding to the performance in the WTM of rats tested in presence of the tone, and the performance of rats treated with salicylate, or salicylate + vehicle, in the absence of the tone; the other, “no-tone”, corresponding to the performance in the WTM of untreated rats and of salicylate + ifenprodil rats, tested in the absence of the tone. Each rat could hence be designated as “no-tone” or “tone” on the basis of its individual performance. In addition, a complementary measure could also be recorded, that is, the number of rats within a given subgroup of rats assigned on the basis of the above individual criterion, which selected the arm associated with the tone as the first choice. Rats displaying the “tone” behavior in the absence of exogenous tone were hence considered to suffer from tinnitus. Rats were designated to the “tone” group if in the test they spent in the tone arm not more than 2 SD (*P* > .05) below the mean time spent in the tone arm by untreated rats tested in the presence of the tone. In contrast, animals were designated to the “no-tone” group if in the test in the absence of the tone they spent in the tone arm not more than 2 SD above the mean time spent by untreated rats tested in the absence of the tone in the tone arm (Figure 2(b)). In the following experiments, none of the animals displayed a behavior which did not fit into either the “tone” or the “no-tone” groups as defined above.

Having established the aforementioned criteria, we proceeded to test the effect of noise overexposure on the induction of long-term tinnitus. Whereas salicylate is known to produce relatively short-term, reversible tinnitus [20], noise overexposure is known to be able to induce long-term, irreversible tinnitus. However, unlike salicylate treatment, the effectiveness of noise overexposure in inducing tinnitus is less predictable [21]. In the noise overexposure experiments, animals were subjected to an intense overexposure of a 6 kHz pure-tone. Noise-induced tinnitus is often characterized by having rather high frequency [6, 22]. Furthermore, the frequency of noise-induced tinnitus is thought to be slightly higher than the frequency of the sound eliciting the tinnitus [23]. Hence, using a frequency of 6 kHz to elicit tinnitus allowed us to analyze tinnitus with a frequency of around 10 kHz. Given that 10 kHz is the best frequency in rat auditory perception, detection of tinnitus with this frequency should be more sensitive [7]. However, to confirm this difference between the frequency of noise overexposure and the expected frequency of tinnitus, an additional group of animals was trained using a 6 kHz pure-tone as the CS, before being subjected to noise overexposure.

**Figure 3:**
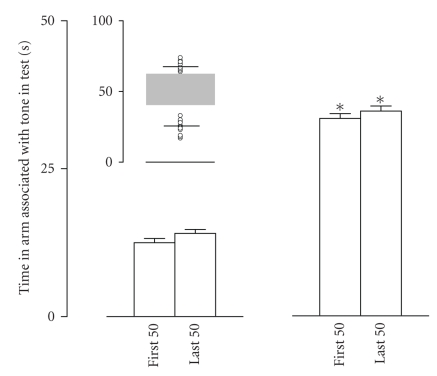
*Induction of tinnitus by sound overexposure.* The time spent in the tone arm by the conditioned rats tested in the absence of the tone, 2 weeks after the sound overexposure. *Inset*: raw data of WTM performance of the conditioned rats that underwent sound overexposure (*n* = 26). These rats segregated into two populations, based on the criteria depicted in Figure 2(b): in one population (*n* = 14), all the rats behave as if they are not experiencing the tone, while in the second (*n* = 12), as if they are experiencing the tone in its absence (for the grey zone, see Figure 2). *Insert:* the horizontal line in each group is the mean of that group; the individual data points are also displayed. *Left and right panels:* time spent by these two populations, respectively, in the tone arm during the first and the last 50 seconds of the test (*P < .01 compared to the other subpopulation, as well as to the conditioned untreated rats tested in the absence of the tone).

Two weeks after noise overexposure, rats conditioned to a pure tone of 6 kHz did not display behavioral evidence of tinnitus in the WTM test. Rats in this group spent 14.3±0.8 seconds during the first 50 seconds and 15.5±0.6 seconds during the last 50 seconds of the test in the tone arm (*n* = 8, not significantly different from the conditioned animals tested in the absence of the tone). In contrast, two weeks after noise overexposure, rats conditioned to the 10 kHz (*n* = 26) tone did not represent a uniform and homogeneous population. Rather, as demonstrated by the individual performance data portrayed in Figure 3, they segregated into two subgroups based on the aforementioned tinnitus-detection criteria. Rats in the first subgroup (*n* = 14) behaved like untreated rats tested in the absence of the tone (see Figure 3). These rats spent 12.5±0.7 seconds during the first 50 seconds and 14.1±0.7 seconds during the last 50 seconds of the test in the tone arm (not significantly different from untreated conditioned rats tested in the absence of the tone). Rats in this group selected the arm associated with the tone as their first choice in only 3/14 of the cases. Rats from the second subgroup (*n* = 12) displayed a response similar to that expected in presence of the tone (see Figure 3). These rats spent 33.3±0.8 seconds during the first 50 seconds and 34.6±0.9 seconds during the last 50 seconds of the test in the tone arm (different from the conditioned animals tested in the absence of the tone, *P* < .001; not different from the conditioned animals tested in presence of the tone, see Figure 3). Furthermore, they selected the tone arm in 8/12 of the cases, *P* < .01 compared to the first subgroup, Mann-Whitney test. All in all, our data demonstrate that noise overexposure induces long-term tinnitus, but that only about a half of the treated rats develop tinnitus. It is noteworthy that this partial effectiveness is similar to that observed in humans [2, 5].

### 3.3. Molecular mechanisms of noise-induced tinnitus

**Figure 4:**
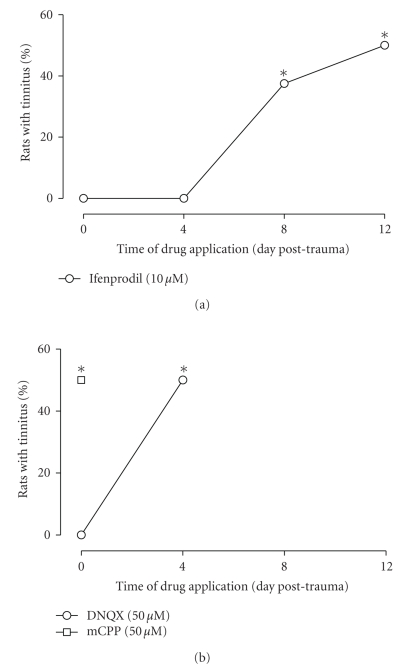
*Effect on tinnitus of cochlear application of glutamatergic antagonists as a function of time after noise overexposure.* Depicted is the proportion of animals experiencing tinnitus, as evident in the WTM test, 2 weeks after sound overexposure. Day 0 corresponds to drug application performed just *before* sound overexposure. (a) *Effect of local cochlear application of ifenprodil as a function of time.* When applied just before sound overexposure, none of the animals displayed tinnitus when tested 2 days later. Similar results were obtained when ifenprodil was applied 4 days after sound overexposure. However, when ifenprodil was applied 8 or 12 days after sound overexposure, about 50% of the rats experienced tinnitus (not different from the proportion observed in the group that underwent sound overexposure, *chisquare* test (*P < .05 compared to the group of animals receiving ifenprodil at day 0, Wilcoxon test)). (b) *Effect of DNQX and mCPP.* When applied just before sound overexposure, DNQX totally prevented the occurrence of tinnitus 2 weeks afterwards. However, when applied 4 days after sound overexposure, no protective effect was evident. Application of mCPP just before sound overexposure did not modify the proportion of rats experiencing tinnitus (*n* = 8 each, *P < .05 compared to the group of animals receiving ifenprodil at Day 0, Wilcoxon test).

Our previous findings on the role of NMDA receptor [7], and particularly the present findings (see above) on the role of the 2B subunit of the NMDA receptor (NR2B) in salicylate-induced tinnitus, have led us to investigate the role of the NR2B in noise-induced tinnitus. Toward that end, we used again the NR2B antagonist ifenprodil. None of the rats receiving local application of ifenprodil just before sound overexposure (*n* = 8) demonstrated behavioral evidence of tinnitus in the WTM (see Figure 4). These rats spent 13.4±0.8 seconds and 13.8±0.8 seconds during the first and the last 50 seconds of the test in the tone arm (not significantly different from the conditioned animals tested in the absence of the tone). Similar results were observed when ifenprodil was applied to the cochlea 4 days after the noise overexposure (see Figure 4(a)). Two weeks after the noise overexposure, none of these rats (*n* = 8) displayed behavioral evidence of tinnitus. They spent 12.9 ± 1 seconds in the first 50 seconds and 13.1±0.6 seconds in the last 50 seconds of the test in the tone arm (not significantly different from the conditioned rats tested in the absence of the tone).

In contrast, when ifenprodil was applied 8 days after noise overexposure, 3 of the 8 rats in the group displayed behavioral evidence of tinnitus according to the individual criteria detailed above (not statistically different from the noise-overexposed group, but statistically different from that observed when ifenprodil was applied just before or 4 days after sound overexposure, *P* < .05). These rats spent 35.7±0.9 seconds and 33.7±0.9 seconds in the first and the last 50 seconds of the test in the tone arm, although the external tone was absent (different from untreated conditioned animals tested in the absence of the tone, *P* < .001; not different from the untreated conditioned animals tested in presence of the tone). All the rats in this subgroup selected the tone arm as their first choice. The other 5 rats spent only 13.8±1.4 seconds during the first 50 seconds and 14.2±0.9 seconds during the last 50 seconds of the test in the tone arm, and four of them selected the no-tone arm as their first choice, in line with the absence of tinnitus in the group (*P* < .05 compared to the group of animals receiving ifenprodil at Day 0, Wilcoxon test).

Half of the rats (4/8) that received ifenprodil 12 days after the sound overexposure demonstrated evidence of tinnitus (see Figure 4(a)). This proportion is not statistically different from the noise-overexposed group, but different from the proportion observed when ifenprodil was applied just before or 4 days after the sound overexposure (*P* < .05). The rats in this subgroup spent 13.5±1.3 seconds and 13.8±1.0 seconds in the tone arm in the first and the last 50 seconds of the test, respectively. This is not significantly different from conditioned intact rats tested in the absence of the tone. Rats in the other subgroup (i.e., the other 4) spent 36.8±1.7 seconds and 34.8±1.0 seconds in the first and the last 50 seconds of the test, respectively, in the tone arm in the absence of the external tone. This is different from the conditioned animals tested in the absence of the tone (*P* < .001) but not different from the conditioned animals tested in presence of the tone.

None of the rats receiving the AMPA receptor antagonist DNQX just before the sound overexposure on Day 0 provided evidence of tinnitus (*n* = 8). In this case, the time spent in the tone arm was 12.8±1.0 seconds and 13.5±1.0 seconds, respectively, for the first and last 50 seconds of the test (not significantly different from untreated conditioned rats tested in the absence of the tone). For rats that received the application of DNQX 4 days after the sound overexposure (*n* = 8), 50% demonstrated evidence of tinnitus (see Figure 4(b); not statistically different from the noise-overexposed group, but different from the proportion observed when DNQX was applied just before the sound overexposure, *P* < .05). Four of these rats spent an average of 36.8±0.5 seconds in the first 50 seconds and 34.3±0.5 in the last 50 seconds of the test in the tone arm (different from conditioned untreated rats tested in the absence of the tone, *P* < .001, not different from conditioned untreated rats tested in presence of the tone). The other 4 rats in this 4-day postexposure DNQX group spent 10.3±0.9 seconds in the first 50 seconds and 13.0±1.4 in the last 50 seconds of the test in the tone arm (not significantly different from the untreated conditioned rats tested in the absence of the tone). None of these animals preferred the tone arm as first choice (*P* < .05 compared to the group of animals receiving ifenprodil at Day 0, and *P* < .05 compared to the group of animals receiving DNQX at Day 0, Wilcoxon test).

Local application of the serotonergic agent mCPP into the cochlea just before noise overexposure did not prevent the occurrence of tinnitus (*n* = 8, see Figure 4(b)). Two weeks after noise overexposure, 50% of these rats displayed tinnitus-like behavior (not statistically different from the noise-overexposed group, but different from the proportion observed when ifenprodil was applied just before, or 4 days after the sound overexposure, *P* < .05). Rats in the subgroup that displayed behavioral evidence of tinnitus: four of these rats spent 37.3±0.9 seconds and 35.5±1.0 seconds in the first and the last 50 seconds of the test in the tone arm in the absence of the tone (different from untreated conditioned rats tested in the absence of the tone, *P* < .001; not different from untreated conditioned rats tested in presence of the tone). All these rats selected the tone arm as their first choice. In contrast, rats in the other subgroup spent 12.3±1.0 seconds and 14.3±0.9 seconds in the first and last 50 seconds of the test in the tone arm (not significantly different from the untreated conditioned rats tested in the absence of the tone), and only one of these rats selected the no-tone arm as the first choice.

## 4. DISCUSSION

It is estimated that about 10% of the adult population in industrialized societies suffer some form of chronic tinnitus [1, 3]. In most cases, it is the consequence of noise trauma, posing a rather widespread and expanding occupational or clubbing hazard. Drug toxicity, a side effect of medications such as salicylates and certain antibiotics, can also cause tinnitus. Over the years, multiple hypotheses have been raised concerning the neuronal locale(s) of the insult, including the peripheral auditory system, the central auditory system, or higher-order, limbic structures [4, 8, 9].

In this study, we set out to investigate neurobiological mechanisms of tinnitus, with the long-term objective of identifying targets for intervention to prevent or ameliorate the pathology. We first developed a new behavioral paradigm to measure tinnitus in the rat. Using this paradigm, we demonstrated that local cochlear application of ifenprodil, an antagonist of the 2B subunit of the NMDA receptor (NR2B), prevents the long-term occurrence of noise-induced tinnitus, suggesting that the 2B subunit of the NMDA receptor complex (NR2B) may be critically involved in the induction of tinnitus by salicylate. By analyzing the time-window of sensibility of noise-induced tinnitus to ifenprodil, we then discovered that long-term tinnitus undergoes a consolidation period of several days, during which tinnitus could be abated by blockade of NR2B in the cochlea. These results broaden our understanding of tinnitus and pave the way to the development of novel methods to prevent or ameliorate it. Furthermore, these data also reflect on the notion of consolidation in neural plasticity in general.

### 4.1. Perception of tinnitus in rats

Designing a behavioral paradigm to determine tinnitus in animals is highly challenging. In the test described here, the expectation was that animals with tinnitus would behave as though they hear a tone even in its absence. This was indeed proven to be the case: in a task involving conditioned tone arm association, treated animals expected to have tinnitus (following either salicylate treatment or noise overexposure) behaved in the absence of a tone as if they were hearing it. This new behavioral paradigm allowed us to define a criterion to decide whether freely moving rats are experiencing tinnitus, and validate it using salicylate treatment under conditions that are established to induce 100% tinnitus.

The new protocol also provided us with the ability to dissect some perceptual attributes of tinnitus. By separating data obtained during the test interval into two 50-second segments, we were able to show difference in the behavior in the presence of the tone between salicylate-, noise-treated, and control rats. There was a decrease over the test in the time that the salicylate rats spent in the tone arm, which was not observed in the group of noise-exposed animals. This could be explained by assuming that salicylate rats underwent some perceptual depreciation during the test, realizing that salicylate-induced tinnitus does not really “sound” as the tone. An alternative interpretation of this difference could be that the tinnitus percept induced by salicylate changes over time. In the case of salicylate, tinnitus percept could have been modulated by other factors, such as the stress of not finding the platform. However, such a modulation is unlikely to have occurred, since animals presenting noise-induced tinnitus do not display such behavior. The fact that salicylate-induced tinnitus and noise-induced tinnitus differ in their perceptual characteristics is of major importance. Given the fact that the vast majority of animal data in the field of tinnitus research was obtained using salicylate to induce tinnitus, caution should be practiced in generalizing the perceptual and mechanistic conclusions to noise-induced tinnitus. Some authors indeed reported particular behavioral aspects related to noise-induced tinnitus [21], but a direct comparison of the validity of salicylate-induced tinnitus as a relevant model for noise-induced tinnitus was still lacking. Our study reinforces the almost tautological conclusion that the best model of noise-induced tinnitus is noise-induced tinnitus.

Having said that, the molecular mechanisms of salicylate-induced tinnitus are of marked importance. Both in vitro and in vivo data indicate that salicylate interferes in the cochlea with glutamatergic neurotransmission, particularly by selectively amplifying NMDA-mediated responses [24, 25]. Furthermore, pharmacological experiments have shown that this pathway lies at the source of salicylate-induced tinnitus [7]. The detailed mechanisms, however, are yet to be deciphered. We add to the elucidation of these mechanisms by suggesting here that the 2B subunit of the NMDA receptor is particularly involved in this process.

### 4.2. Molecular bases of noise-induced tinnitus and therapeutic potential

In contrast with noise-induced tinnitus, salicylate-induced tinnitus only lasts for a short period of time [7, 20]. Noise-induced tinnitus is therefore of a greater clinical significance. Whereas under the conditions used in this study, salicylate treatment induced tinnitus in all treated animals (as expected), the severe noise insult yielded tinnitus in only part of the treated group. This probabilistic characteristic of noise-induced tinnitus is well documented in humans, and has also been suggested in animals [21].

In considering potential pharmacopeia for noise-induced tinnitus, the first synapse of the auditory pathways (the synapse between inner hair cells and primary auditory neurons) is an appealing target. Indeed, in this study, when locally applied during the first 4 days following the noise overexposure, the NR2B-containing NMDA receptor antagonist ifenprodil was able to completely abate long-term noise-induced tinnitus.

Taking into account the fact that the synapses between inner hair cells and primary auditory neurons are glutamatergic [26], the question arises whether the NMDA receptor antagonist ifenprodil acts to repair the damage or protects against glutamate-induced excitotoxicity. Glutamate-induced excitotoxicity is often associated with overactivity of AMPA receptors, but NMDA receptors have also been implicated [27, 28]. Whereas the local application of ifenprodil led to abolishment of tinnitus even when applied 4 days after the noise overexposure, local application of the AMPA receptor antagonist DNQX had an effect only immediately before the noise overexposure. The fact that local application of DNQX failed to decrease the ratio of occurrence of tinnitus after exposure to an acoustic trauma when the application was done 4 days after the trauma shows that AMPA involvement in noise-induced damage mainly occurs at the first stage after the insult, that is, during the period of the postulated response to excitotoxicity.

The lack of effect of the serotonergic agent mCPP just before the acoustic trauma on the expression of tinnitus two weeks afterwards argues against nonspecific effects of the surgery or the dilution of endogenous perilymph with the vehicle. Furthermore, to validate the specificity of the effect, we also locally applied a drug that is irrelevant to the genesis of salicylate-induced tinnitus: the serotonergic agent mCPP [12]. Incidentally, those results also ruled out a putative role of serotonin in the generation of noise-induced tinnitus.

### 4.2. Generality of memory consolidation windows

It is noteworthy that the postinsult time window during which the NMDA receptor antagonist can abate tinnitus is limited to a few days only. A transient time-window of susceptibility to blockers of experience-dependent plasticity is a universal property of memory systems, and is referred to as consolidation [29, 30]. Furthermore, though the NMDA receptor was considered to play a role in memory encoding only [31], evidence exists that it may play a key role in memory consolidation as well [32, 33]. The exact mechanisms of the NMDA receptor blockade in tinnitus notwithstanding (see remark on repair or protection above) the phenomenon of a transient sensitivity window during which an experience-dependent neuronal change can be abated may not be a hallmark of learning and memory systems only, and apply as well to experience-dependent modifications that are not construed as learning. Indeed, tinnitus could be regarded as experience-dependent modification in a neuronal system, which may share mechanisms with memory systems even at the earliest station in which the insult leaves an imprint.

This conclusion is not only of a conceptual nature, as it bears also upon the potential mechanisms of tinnitus. Multiple hypotheses have been raised concerning the critical locale of the tinnitus-induced insult, including the peripheral auditory system, the central auditory system, or higher-order brain structures [4, 9]. The possibility cannot be excluded that tinnitus involves several or all of these locales, and that they are recruited over time, in a process that resembles memory systems consolidation, that is, the translocation of an engram from one location to another and its distribution over multiple loci [30, 34]. If this is the case, clearly, the effectiveness of the treatment of long-term, noise-induced tinnitus is expected to decline over time after the insult. The finding that local application of a specific receptor antagonist into the first station of the auditory system is an effective treatment in the first days after the insult, under conditions that should minimize systemic side effects, is therefore of marked potential clinical implication.

## Figures and Tables

**Table 1:** *Experimental groups*. When not specified, conditioning was performed using a sound of 10 kHz, as described in Section 2. Salicylate refers to a daily injection of salicylate (300/mg/kg) between Day 12 and Day 15. Acoustic trauma refers to the day when the acoustic overexposure was performed. The name of a pharmacological agent means that this agent was locally applied within cochlear fluids at the indicated day. When a drug was applied at Day 0, the application was performed just before the sound exposure.

Day −4 to Day −2	Day 0	Day 4	Day 8	Day 12	Day 15	*n*
*Control unconditioned animals*						
					Test (silence)	8
					Test (sound 10 kHz)	8
				Salicylate	Test (silence)	8

*Calibration experiments*						
Conditioning					Test (silence)	8
Conditioning					Test (sound 10 kHz)	8

*Salicylate experiments*						
Conditioning				Salicylate	Test (silence)	8
Conditioning				Artificial perilymph Salicylate	Test (silence)	8
Conditioning				Ifenprodil (10 *μ*M) Salicylate	Test (silence)	8

*Acoustic trauma experiments*						
Conditioning	Acoustic trauma				Test (silence)	26
Conditioning (6 kHz)	Acoustic trauma				Test (silence)	8

*Pharmacological experiments*						
Conditioning	Ifenprodil (10 *μ*M) Acoustic trauma				Test (silence)	8
Conditioning	Acoustic trauma	Ifenprodil (10 *μ*M)			Test (silence)	8
Conditioning	Acoustic trauma		Ifenprodil (10 *μ*M)		Test (silence)	8
Conditioning	Acoustic trauma			Ifenprodil (10 *μ*M)	Test (silence)	8
Conditioning	DNQX (50 *μ*M) Acoustic trauma				Test (silence)	8
Conditioning	Acoustic trauma	DNQX (50 *μ*M)			Test (silence)	8
Conditioning	mCPP (50 *μ*M) Acoustic trauma				Test (silence)	8

## References

[1] Coles R, Shulman A Epidemiology of tinnitus: (1) prevalence.

[2] Dieroff HG, Meissner W (1987). Prevalence of tinnitus in noise-induced hearing loss.

[3] Goebel G (1995). Fortschritte bei der verhaltensmedizinischen diagnostik und behandlung quälender chronischer ohrgeräusche. *Oto-Rhino-Laryngologia Nova*.

[4] Guitton MJ (2006). Tinnitus and anxiety: more than meets the ear. *Current Psychiatry Reviews*.

[5] Chung DY, Gannon RP, Mason K (1984). Factors affecting the prevalence of tinnitus. *Audiology*.

[6] Nicolas-Puel C, Faulconbridge RL, Guitton M, Puel J-L, Mondain M, Uziel A (2002). Characteristics of tinnitus and etiology of associated hearing loss: a study of 123 patients. *International Tinnitus Journal*.

[7] Guitton MJ, Caston J, Ruel J, Johnson RM, Pujol R, Puel J-L (2003). Salicylate induces tinnitus through activation of cochlear NMDA receptors. *Journal of Neuroscience*.

[8] Eggermont JJ, Roberts LE (2004). The neuroscience of tinnitus. *Trends in Neurosciences*.

[9] Eggermont JJ (2005). Tinnitus: neurobiological substrates. *Drug Discovery Today*.

[10] Kaltenbach JA, Zhang J, Finlayson P (2005). Tinnitus as a plastic phenomenon and its possible neural underpinnings in the dorsal cochlear nucleus. *Hearing Research*.

[11] Obstreicher E, Arnold W, Ehrenberger K, Felix D (1999). New approaches for inner ear therapy with glutamate antagonists. *Acta Oto-Laryngologica*.

[12] Guitton MJ, Pujol R, Puel J-L (2005). m-chlorophenylpiperazine exacerbates perception of salicylate-induced tinnitus in rats. *European Journal of Neuroscience*.

[13] Jastreboff PJ, Brennan JF, Coleman JK, Sasaki CT (1988). Phantom auditory sensation in rats: an animal model for tinnitus. *Behavioral Neuroscience*.

[14] Monyer H, Burnashev N, Laurie DJ, Sakmann B, Seeburg PH (1994). Developmental and regional expression in the rat brain and functional properties of four NMDA receptors. *Neuron*.

[15] Nicoll RA, Malenka RC (1995). Contrasting properties of two forms of long-term potentiation in the hippocampus. *Nature*.

[16] Rosenblum K, Dudai Y, Richter-Levin G (1996). Long-term potentiation increases tyrosine phosphorylation of the N-methyl-D-aspartate receptor subunit 2B in rat dentate gyrus in vivo. *Proceedings of the National Academy of Sciences of the United States of America*.

[17] Rosenblum K, Berman DE, Hazvi S, Lamprecht R, Dudai Y (1997). NMDA receptor and the tyrosine phosphorylation of its 2B subunit in taste learning in the rat insular cortex. *Journal of Neuroscience*.

[18] Tang Y-P, Shimizu E, Dube GR (1999). Genetic enhancement of learning and memory in mice. *Nature*.

[19] Wang J, Dib M, Lenoir M (2002). Riluzole rescues cochlear sensory cells from acoustic trauma in the guinea-pig. *Neuroscience*.

[20] Cazals Y (2000). Auditory sensori-neural alterations induced by salicylate. *Progress in Neurobiology*.

[21] Heffner HE, Harrington IA (2002). Tinnitus in hamsters following exposure to intense sound. *Hearing Research*.

[22] Cahani M, Paul G, Shahar A (1983). Tinnitus pitch and acoustic trauma. *Audiology*.

[23] Loeb M, Smith RP (1967). Relation of induced tinnitus to physical characteristics of the inducing stimuli. *Journal of the Acoustical Society of America*.

[24] Peng B-G, Chen S, Lin X (2003). Aspirin selectively augmented N-methyl-D-aspartate types of glutamate responses in cultured spiral ganglion neurons of mice. *Neuroscience Letters*.

[25] Guitton MJ, Puel J-L (2004). Cochlear NMDA receptors and tinnitus. *Audiological Medicine*.

[26] Glowatzki E, Fuchs PA (2002). Transmitter release at the hair cell ribbon synapse. *Nature Neuroscience*.

[27] Chen Q, Surmeier DJ, Reiner A (1999). NMDA and non-NMDA receptor-mediated excitotoxicity are potentiated in cultured striatal neurons by prior chronic depolarization. *Experimental Neurology*.

[28] Rocha M, Martins RAP, Linden R (1999). Activation of NMDA receptors protects against glutamate neurotoxicity in the retina: evidence for the involvement of neurotrophins. *Brain Research*.

[29] McGaugh JL (2000). A century of consolidation. *Science*.

[30] Dudai Y (2004). The neurobiology of consolidations, or, how stable is the engram?. *Annual Review of Psychology*.

[31] Day M, Morris RG (2001). Memory consolidation and NMDA receptors: discrepancy between genetic and pharmacological approaches. *Science*.

[32] Shimizu E, Tang Y-P, Rampon C, Tsien JZ (2000). NMDA receptor-dependent synaptic reinforcement as a crucial process for memory consolidation. *Science*.

[33] Takehara-Nishiuchi K, Nakao K, Kawahara S, Matsuki N, Kirino Y (2006). Systems consolidation requires postlearning activation of NMDA receptors in the medial prefrontal cortex in trace eyeblink conditioning. *Journal of Neuroscience*.

[34] Dudai Y, Morris RGM (2000). To consolidate or not to consolidate: what are the questions?. *Brain, Perception, Memory. Advances in Cognitive Sciences*.

